# Laser Capture Microdissection in the Spatial Analysis of Epigenetic Modifications in Skin: A Comprehensive Review

**DOI:** 10.1155/2022/4127238

**Published:** 2022-02-09

**Authors:** Theja Bhamidipati, Mithun Sinha, Chandan K. Sen, Kanhaiya Singh

**Affiliations:** ^1^Kansas City University, 1750 Independence Avenue, Kansas City, MO, USA; ^2^Indiana Center for Regenerative Medicine and Engineering, Department of Surgery, Indiana University School of Medicine, Indianapolis, IN, USA

## Abstract

Each cell in the body contains an intricate regulation for the expression of its relevant DNA. While every cell in a multicellular organism contains identical DNA, each tissue-specific cell expresses a different set of active genes. This organizational property exists in a paradigm that is largely controlled by forces external to the DNA sequence *via* epigenetic regulation. DNA methylation and chromatin modifications represent some of the classical epigenetic modifications that control gene expression. Complex tissues like skin consist of heterogeneous cell types that are spatially distributed and mixed. Furthermore, each individual skin cell has a unique response to physiological and pathological cues. As such, it is difficult to classify skin tissue as homogenous across all cell types and across different environmental exposures. Therefore, it would be prudent to isolate targeted tissue elements prior to any molecular analysis to avoid a possibility of confounding the sample with unwanted cell types. Laser capture microdissection (LCM) is a powerful technique used to isolate a targeted cell group with extreme microscopic precision. LCM presents itself as a solution to tackling the problem of tissue heterogeneity in molecular analysis. This review will cover an overview of LCM technology, the principals surrounding its application, and benefits of its application to the newly defined field of epigenomics, in particular of cutaneous pathology. This presents a comprehensive review about LCM and its use in the spatial analysis of skin epigenetics. Within the realm of skin pathology, this ability to isolate tissues under specific environmental stresses, such as oxidative stress, allows a far more focused investigation.

## 1. Introduction

**Figure 1:**
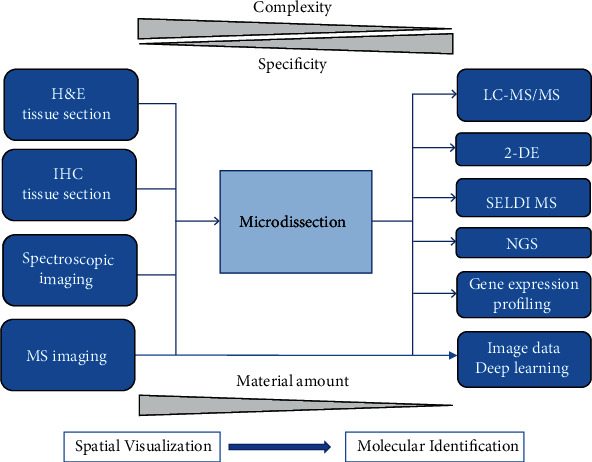
Many downstream analysis relies on the fundamental principle behind microdissection. An inappropriate tissue dissection would then be implicated in lower yield results and contamination. Reproduced under the terms of the Creative Commons CC BY license published by John Wiley and sons. The following original report is credited: von Eggeling and Hoffmann [4].

The premise behind cellular analysis rests largely on the assumption that tissue samples consist of homogenous cells. Historically, this process has disregarded the spatial aspect of the tissue in question. More so, the assumption of tissue homogeneity ignores the fact that identical biologic stressors can cause different reactions in different cell types [1]. Thus, having the ability to specify both spatial and cellular criteria, such as in a biopsy of a suspicious lesion, can allow the identification of molecular pathways that can distinguish between a metastatic process and a more benign etiology [2]. The current dogma behind these molecular pathways is that they rely heavily on heterogeneous cell populations due to their reliance on low abundance molecules, their spatial nature, and their interrelation [3]. Epigenetic modification, in particular, must use downstream cascades in order to properly affect gene transcription simply due to the extracellular nature of the modification. Of note, these downstream cascades are highly variable among different tissue types and even among similar tissues that exist in different environments. When focusing on epigenetic analysis, processes such as CpG methylation are extremely specific to parts of the DNA sequence. Properly isolating target cells and tissue would thus be even more important in the analysis of epigenomics when compared to more standard analytic tools that analyze protein structure or RNA content. The applications of this “microdissection” can be applied to nearly every field for molecular investigation including proteomics and transcriptomics [4] (Figure 1).

As the DNA sequence has become more understood with the completion of the Human Genome Project in 2003, there has been a shifting focus from solely identifying the genetic component of many diseases to detecting the progression of these same diseases [5–7]. High-risk cancerous tissue, for example, can be analyzed for certain epigenetic modifications which can be used as a proxy for potential malignant transformation [8]. The ability to identify potential malignancy offers avenues for early intervention to prevent morbidity and mortality. The analysis of these types of tissue has historically been done using techniques such as Southern blot analysis and polymerase chain reaction. However, there is a growing need for a new generation of sequencing techniques that can fine-tune the selection of tissue in regard to spatial location and pathological stresses.

When used in unison, isolation of discrete cell types/tissues and new sequencing platforms can create a genome-wide profile that has the power to unveil new information that can challenge previous dogmas. For example, Li et al. demonstrated the necessity for precise tissue sampling by showing that the difference between young and old skin did in fact have different dermal molecular expression in the dermis [9]. This difference was previously ignored because tissue samples rarely were separated from their epidermal components. Clearly, the ability to differentiate between seemingly insignificant parts of the same tissue can have profound implications.

Laser microbeam microdissection (LMM) and its successor laser capture microdissection (LCM) represent the newest technologies meant to overcome the inherent heterogeneity of tissue samples. By using the principles behind ultraviolet and infrared laser, the specificity of cell samples taken in situ is orders of magnitude greater. Thus, further downstream analysis, such as in epigenetics, becomes far more sensitive and specific.

## 2. Laser Capture Microdissection

### 2.1. Overview

**Figure 2:**
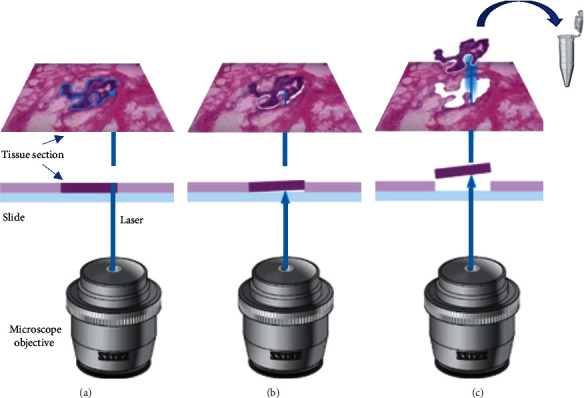
Inverted microscope used in laser microbeam (LMM) and laser capture microdissection (LCM) deploying ultraviolet or infrared laser pulses to target specific tissue sections. The laser-based microdissection is performed in three steps. (a) The laser coupled in the objective separated the desired area from the surrounding tissue. (b) The capture of the marked sample is then initiated using a single laser pulse. (c) The marked sample is then transferred to the desired capture device for downstream analyses. Reproduced under the terms of the Creative Commons CC BY license published by John Wiley and sons. The following original report is credited: von Eggeling and Hoffmann [4].

The use of applying focused lasers to isolate target tissues first began in the early 20^th^ century but has advanced in recent years [1]. The initial need for a faster process than manual microdissection led to the introduction of microbeams. LMM focused primarily on using an ultraviolet laser on membrane-mounted sections of tissue. The membrane offers good optical quality without interfering with the downstream analysis. Pulses of ultraviolet lasers cause local dissection of the membrane-tissue complex due to photolysis [10]. The dissected tissue would then be taken out and used for further analysis. However, while LMM offered quality dissection with little dispersion of the tissue, it required a great deal of dexterity and is time-consuming. LCM was then introduced by the National Cancer Institute of the National Institutes of Health in Bethesda as the newest generation of laser capture technology. Within the past decade, it has been used to isolate tumor cells, neurons, virus-infected cells, and even specific organelles such as nuclei [11, 12].  Figure 2 pictorially represents the ability of LCM to isolate specific tissue elements [4].

In 1976, Isenberg et al. were the first to use this laser technology but in surgery [13]. It required massive space to dissect out tissue subpopulations. In response, LCM was devised by Emmert-Buck and their team [13]. They recognized a need for a system for efficient dissection of cells of solid tumors to fully utilize analytical technologies. This process quickly moved to a commercial platform by Arcturus Engineering under the name Veritas Systems (Mountain View, CA) [11, 14]. PALM Microbeam system (Carl Zeiss MicroImaging GmbH, Bernried, Germany) and the Leica LMD6000 (Leica Microsystems Inc., Bannockburn, IL, USA) further commercialized the technology making its use ubiquitous in researchers' labs across the world.

### 2.2. Components of LCM

**Figure 3:**
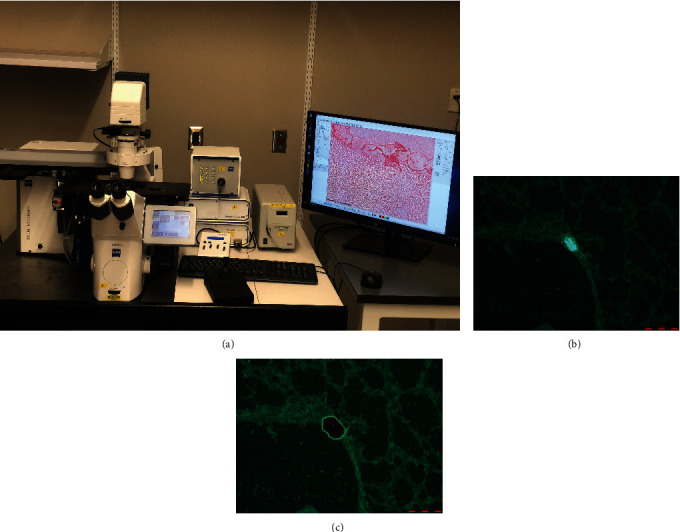
(a) Zeiss PALM Microbeam Laser Microdissection System located at the Indiana Center for Regenerative Medicine and Engineering, Indiana University. Prototypical laser setup showcases the components of LCM. (b) Before and (c) after laser capture microdissection of lung tissue sample done under fluoroscopy. Photographs showing of a lung section before and after images during capture of a cluster of fluorescent neuroendocrine cells, known as a neuroepithelial body. (b, c) Reproduced with permission from John Wiley and sons. The following original report was credited: Jensen [18].

The components for a LCM are (i) visualization of target cells using traditional microscopy, (ii) having the ability to transfer photoenergy to either a polymer-cell composite or directly to photolyze tissue, and (iii) being able to isolate and remove the target sample. This is done with the use of an inverted microscope, an infrared laser, control unit for the laser, a control mechanism for the microscope stage, a digital camera, and a monitor for target visualization [14]. A prototypical setup for large-scale microdissection is shown is Figure 3. Two large classes of LCM that exist are infrared and ultraviolet. Infrared LCM (IR-LCM) primarily uses large wavelength light at approximately 810 nm while ultraviolet LCM (UV-LCM) uses a shorter wavelength at approximately 355 nm [15]. While IR-LCM utilizes photoenergy to melt polymer caps in order to isolate tissue samples, UV-LCM directly photoablates the tissues around cells of interest. The differences and advantages between the two will be discussed in next sections; however, more modern systems are relying on combination of IR and UV-LCM such as the Arcturus XT (Thermo Fisher Scientific).

### 2.3. Infrared LCM (IR-LCM)

Emmert-Buck and coworkers in 1996 at the National Institutes of Health developed an IR-LCM system [13]. By using very short pulses of infrared lasers with wavelengths of about 810 nm, the IR-LCM system is based on a thin thermoplastic film over the tissue section. The IR beams can melt the polymers overlying the collected tissue sample. This creates a tissue-polymer complex that can isolate target cells. More so, the thermofilm functions as a barrier to the damage from the light preserving the tissue [16]. The focused pulse of IR directed at the thermofilm causes a conformational change and adherence of the tissue to the polymer [17]. At the end of the dissection, cells of interest that are adhered to the film can be detached and transferred for analysis. However, due to this thin polymer, there is a risk of the sample becoming attached to the adhesive film running the risk for contamination. More so, this process is heavily user dependent with operator skill being a limiting factor.

### 2.4. Ultraviolet LCM (UV-LCM)

Using smaller wavelength light of about 355 nm, UV-LCM is able to more specifically ablate tissue adjacent to the cells of interest [17]. First developed by Schutze and Lahr in 1998, this platform uses tissue that has been mounted directly on a membrane. Direct visualization by microscopy then allows direct isolation of cell populations without adjacent unwanted tissue [13, 16]. When compared to its IR counterpart, UV avoids the issue surrounding adhering of unwanted cells due to the ability to directly photoablate the sample. More so, the smaller wavelength used in this system allows for more precise beam focus around smaller cells or even organelles. The target cells are then retrieved via photonic pressure or gravity which launches them into the collector cap [17].

### 2.5. Advantages and Limitations of LCM

The advantages of LCM stem from the ability to isolate very small and spatially unique cellular elements. Depending on the laser size and tissue sample, thousands of unique cellular elements can be collected in a fraction of the time that manual microdissection would require. This notion brings up the next advantage of LCM which is the wide-scale application of a fast and easy-to-use technology that does not require substantial manual dexterity or training. Jensen showcased how sensitive tissue from lung biopsies could be easily identified and dissected using LCM [18] (Figure 3). In fact, many LCM microscopes are easily adjusted to correspond with the skill of the operator. When comparing LCM to more conventional techniques, it is important to examine the quality of the tissue samples collected. In 1999, Banks et al. found no gross changes in the protein profiles [19]. The worry about lower quality dissection samples thus does not limit the implementation of LCM in cell and tissue analysis.

However, there are noticeable limitations of LCM which primarily reflect the limitations of microdissection. The narrow band of wavelength offers a physical limit on how small the dissection can be done on subcellular structures [20]. Another issue that is not only seen in LCM is the worry about optical resolution of tissue. Samples in LCM do not require the use of a cover slip. Thus, dehydrated tissue without a coverslip can make precise microdissection difficult. Investigators can however use special staining to highlight the tissue target in question [21]. And while LCM does offer an easier operator learning curve, the physical removal of cells from the slide is operator dependent. This event usually occurs with frozen sections and can be avoided with paraffin wax sections. Luo et al. demonstrate the optimal transfer conditions of frozen tissue sections with specific stains [22]. These immunohistochemistry stains offer their own set of problems. For example, eosin can interfere with certain 2D-Page Gel electrophoresis [23].

However, even when taking into consideration the limitations of laser capture microdissection, the speed, easy handling, and precision make this modality the ideal tool for collection of large amounts of specific tissue and cells.

## 3. Applications of LCM to Epigenetic Studies

### 3.1. Overview

**Figure 4:**
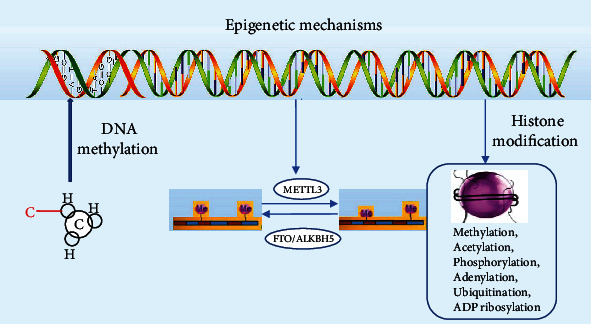
Modification to DNA or RNA through methylation or histone modifications changes the storage and packaging of DNA. Downstream, this can limit transcription. DNA methylation is one of the most studied epigenetic mechanisms, which occurs on CpG islands located in different repetitive genome regions or, more commonly, in promoter regions. Other epigenetic mechanisms known as histone modifications mainly include the methylation, ubiquitylation, acetylation, sumoylation, and phosphorylation of the histone tails. N6-Methyladenosine (m6A) is the most common mRNA modification. m6A modification is conducted by its “readers,” “erasers,” and “writers” to remove, add, or preferentially bind to m6A. m6A methylation occurs at once after pre-mRNA transcription by METTL3-containing methyltransferases, while the demethylation of m6A is by fat mass and obesity associated (FTO) and alpha-ketoglutarate-dependent dioxygenase AlkB homolog 5 (ALKBH5). Reproduced with permission from John Wiley and sons. The following original report was credited: Sang and Deng [26].

**Figure 5:**
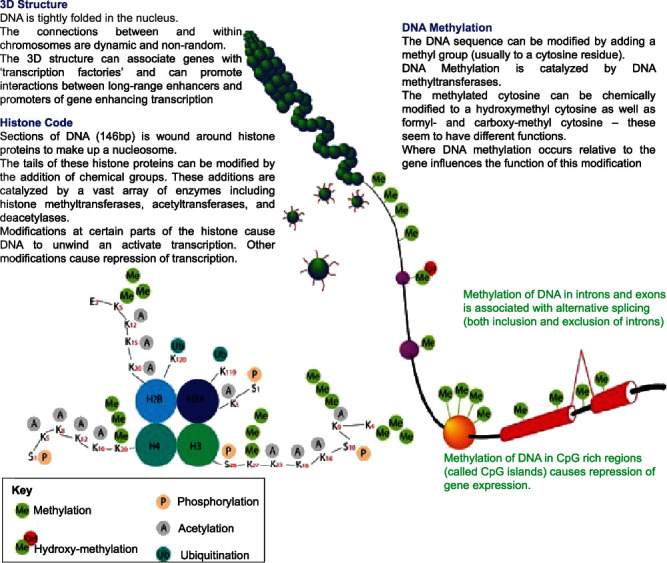
Epigenetic modifications can alter the spatial arrangement of DNA. This rearrangement changes the ability for transcription factors and other modifiers to access the DNA sequence. Reproduced with permission from John Wiley and sons. The following original report was credited: Duncan et al. [27].

Epigenetic modification is the broad-encompassing term that denotes hereditary inheritance that occurs outside of the DNA sequence [24]. These modifications mostly target the storage activation and inactivation of DNA. The three prototypical examples include (i) DNA methylation, (ii) histone modifications, and (iii) chromatin remodeling [25]. As outlined in Figures 4 and 5, these modifications have the ability to change the ability of DNA to be transcribed [26, 27].

**Figure 6:**
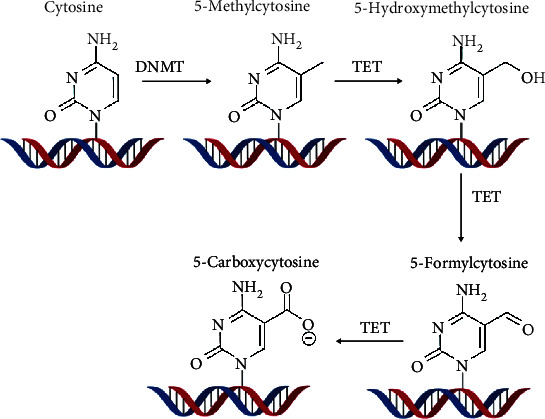
Both DNMT and TET enzymes have elaborated roles in the methylation of DNA. Their clinical significance ranges from solid cell tumors to hematologic malignancies. Reproduced with permission from John Wiley and sons. The following original report was credited: Beyer et al. [33].

DNA methylation is a silencing tool associated with cytosine-guanine dinucleotide repeats found primarily on gene promoters. Recruitment of these additional methyl (CH3) groups to the cytosine base forms a genetically stable carbon-carbon bond resulting in the formation of 5-methylcytosine (5mc) [28]. CpG methylation at these sites on promoters has been associated with gene silencing [28–30]. The CpG methylation events are carried out by DNA methyltransferases (DNMTs) or by the ten-eleven translocation family (TET) [31–33] (Figure 6). In particular, DNMT1, DNMT3A, DNMT3B, TET1, TET2, and TET3 have been shown to be associated with the pathological transcriptional deregulation in many diseases [27]. These diseases range from hematologic malignancies to solid tumor growth. More so, the causes of methylation have been linked environmental and lifestyle factors [34].

On the other hand, histone modifications occur on a larger scale and control gene transcription. As chromatin DNA becomes more condensed and organized, it becomes associated with histone proteins to form a nucleosome [27]. In particular, the four core histone proteins of the nucleosome (H2A, H2B, H3, and H4) are particularly susceptible to modification. Acetylation of these abovementioned histone proteins induces changes in the chromatin configuration that increases gene transcription [27]. Primary histone acetylation is done by acetyltransferases (HAT). On the other hand, deacetylation which is performed by histone deacetylases (HDAC) tightens the conformation of the nucleosome and limits transcription [25].

“Epigenomics” therefore is the study of these epigenetic modifications that occur outside of the genome itself. Of note, there is a strong inheritance pattern exhibited by epigenomics. However, there are also environmental factors that can cause epigenetic changes [35]. This is in stark contrast to proteomics or genomics where the given DNA sequence is all but permanent. The ability to modify environmental variables makes the understanding of epigenomics extremely important for early therapeutic interventions.

### 3.2. Epigenetic Investigation

Advances in the past decade have mushroomed the amount of investigation into genes and genome knowledge (genomics). However, the first paper to study epigenetic modification on microdissected cells was by Wu et al. showing the role of hypermethylation in endometriosis [36]. New pursuits into epigenetic modifications have outlined the extracellular modifications in the heritable transfer of genetic material. These epigenetic modifications are independent of primary DNA sequence changes. More so, epigenetic modifications are strongly linked to environmental cues. As such, the precision offered by LCM becomes even more paramount when different cell types and different physiologic cues can vastly confound downstream analysis.

LCM has been now being extensively used in epigenetic studies. Examination of methylation patterns, for instance, has been long used as a prognostic factor for predicting cancers [2]. LCM functions as an integral part of the cancer epigenetic repertoire. Examining the frequency of epigenetic modifications in cancerous lesions is all but useless if there is no normal sample to compare. When collection is done in situ, it is difficult to differentiate between normal tissue samples and lesions of interest. The issue is further complicated when one considers the spatial heterogeneity of many tissue samples. This heterogeneity is nearly eliminated when the technology of LCM is applied, and precise dissections are able to isolate the sample of interest.

As mentioned earlier, Li et al. (2016) already demonstrated the importance of LCM. *COX2* mRNA expression has been long theorized to play a role in the normal aging process of skin. However, the levels expressed in whole skin preparations did not account for that difference. Only after LCM was used to separate dermal from epidermal constituents did a noticeable difference in mRNA expression via quantitative polymerase chain reaction (qPCR) become visible [37]. Skin in particular lends itself to LCM due to the large role that environmental factors can play. There exists so much variance in sun exposure, gland density, heat/cold tolerance, and wound susceptibility that it is impossible to treat in situ skin samples as homogenous and uniform. Naturally, there is a progression toward using LCM and not just applying it to epigenetic studies but applying it specifically to pathology of the skin.

### 3.3. LCM-Based Epigenetic Studies of the Skin

**Figure 7:**
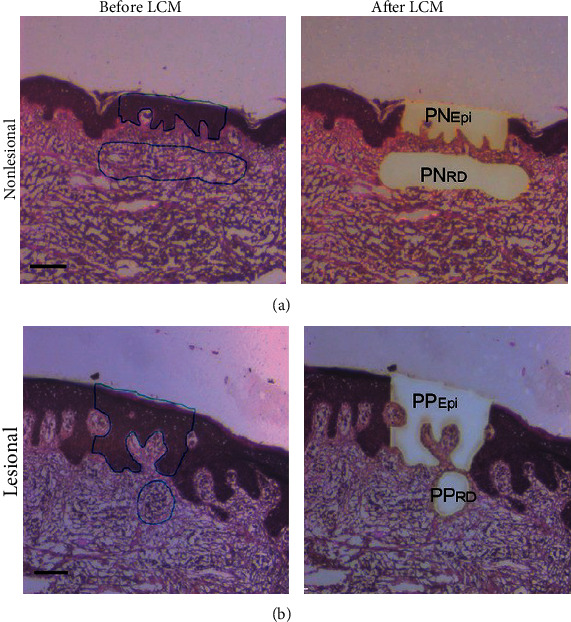
Laser capture microdissection (LCM) can be combined with small RNA sequencing to identify specific miRNA signatures in psoriatic plaque epidermis and dermis. LCM was performed by authors on both (a) normal psoriatic skin and (b) psoriatic plaque samples. Special attention is paid to the cellular differences in the epidermal layers requiring microdissection for downstream analysis. PN_Epi_: normal psoriatic epidermis; PP_RD_: psoriatic plaque dermal inflammatory infiltrates. Reproduced with permission from John Wiley and sons. The following original report was credited: Lovendorf et al. [40].

Cutaneous manifestations of injury are one of the largest areas for epigenetic investigation. The principles surrounding wound injury and healing revolve around the interplay of genetic and environmental factors. Even minor changes in the environment, such as sun exposure, can create an entirely different milieu for the wound to develop. As such, any investigation into the causality of wound healing will revolve around the role of epigenetics [38]. However, the intrinsic variability surrounding human cutaneous wounds makes the isolation of target cells extremely difficult. In situ biopsy of the tissue such as via punch biopsy or excisional biopsy runs the risk introducing unwanted heterogeneity to the tissue sample [39]. Even more, the epidermal and dermal layers of the skin contain a vast array of cells that grossly differ from each other. Thus, the role of LCM becomes paramount in isolating purely pathologic tissue.  Figure 7 demonstrates pictorially how LCM can target specific areas of the skin and avoid unwanted cell types [40].

Biopsies collected from clinical human cutaneous wounds are highly heterogeneous in cellular composition [38]. Furthermore, the composition of the tissue varies based on collection procedure complicating comparison of results derived from tissue homogenates. Thus, the utility of such tissue material is primarily limited to histological studies.

Nothing exemplifies the persistent nature of cutaneous disorders more than that of chronic diabetic ulcers (DCU). DCUs represent the intersection between poor wound repair and specific environmental factors that can affect the progression of proper healing. Nie et al. recently reviewed the role of microRNAs (miRNAs) in nearly all aspects of skin healing including angiogenesis, epithelialization, and tissue remodeling [41]. These factors create a triad of interest when trying to understand the poor healing associated with DCUs. Investigators found multiple distinct pathways regulating miRNAs during chronic wound healing including the Wnt/B-catenin pathway, NF-*κ*B pathway, and PI3K/AKT/mTOR pathway. In particular, the epigenetic regulation of miRNA promoters has been shown to be a determinant of angiogenesis [42]. The role of angiogenesis thus requires further investigation as it is a hallmark of proper wound healing through the supply of nutrients.

**Figure 8:**
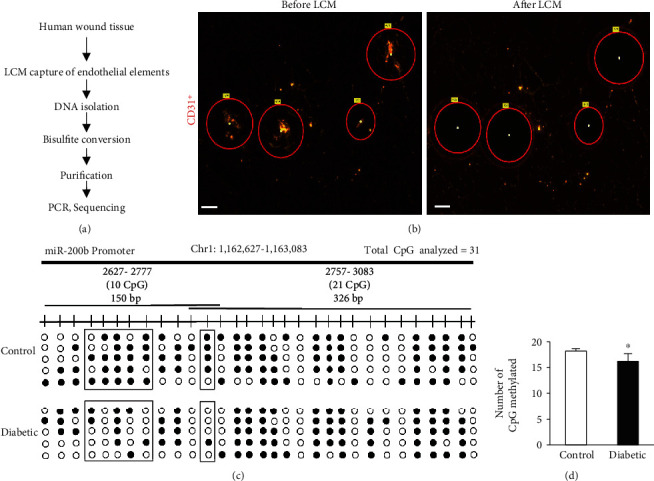
Figure showing connection between microdissection and epigenetic analysis as published by our group [42]. (a) Schematic diagram showing experimental design of miR-200b promoter methylation analysis in endothelial elements collected from human chronic wounds. (b) Representative figure shows the selection of CD31^+^ tissue elements (red) and their collection before and after the laser capture microdissection (LCM). Scale bar, 150 *μ*m. (c, d) Methylation profile and quantitation of methylated CpG islands present in miR-200b promoter in diabetic wounds compared to normoglycemic wounds. Reproduced with permission from the American Society of Gene and Cell Therapy. The following original report was credited: Singh et al. [42].

In order to further understand the role of epigenetics and angiogenesis, Singh et al. utilized laser capture technologies to specifically target wound edges in diabetic patients (Figure 8) and in mice [42]. This tissue was precisely selected for its microenvironment. Downstream analysis of this tissue showed stark differences in the expression of miRNA-200b profiles of diabetic mice as compared to nondiabetic cohorts [42]. This specific capture of tissue would not have been possible with traditional methods where the specific wound edge environment could be isolated.

A further investigation into the poor revascularization factors of diabetic wounds was also performed by the same group in 2019 [43]. This second investigation showed that there is a required process, similar to the epithelial-mesenchymal transition, which must be activated in order for proper wound healing. In particular, transcription factor ZEB1 was implicated to be an activator of this transition process primarily via vascular endothelial growth factor (VEGF) [43]. The mechanism of this pathway was shown to be *via* many miRNAs with special attention focused to miR-200b. Investigators in this report once again used LCM to target regions of human wounds with rich epithelial cell density. The captured cells were then subjected to downstream analysis. The results supported the hypothesis that at the wound edge, low levels of ZEB1 resulted in compromised angiogenesis and delayed wound closure.

The complexity of cell types and samples seen at wound sites makes the specific analysis of epigenetics and other downstream events difficult. LCM offers a new gold standard for the acquisition of tissue samples. The possible implications of having a comprehensive understanding of angiogenesis in cutaneous wounds include the development of therapeutic drugs used for diabetic or other chronic wounds.

### 3.4. LCM to Study miRNA Regulation

miRNAs act as important posttranscriptional regulators of gene expression. One of the factors to make a rational choice for miRNAs as biomarker or drug targets is posed by exactly this intrinsic heterogeneity in miRNA expression between different cell types and intrinsic cellular complexity of an organ or tissue. LCM helps in disentangling tissue heterogeneity, and when combined with RT-qPCR analysis, it can reveal compartment-specific miRNA expression signatures. This helps in linking miRNA signature to histopathology [44]. This approach for spatial miRNA expression analysis in complex tissues enables discovery of disease mechanisms, biomarkers, and drug candidates. Simoes et al. performed LCM on murine oral mucosal wound tissue to identify miRNA signature in the epithelial tissues [45]. Sinha et al. laser captured the wound-site macrophages during the healing phase to identify their plasticity to fibroblast-like cells [46]. The authors observed increased expression of miR-21 in these LCM-captured macrophages which displayed plasticity. Similar studies using miR-21 were reported by Bejerano et al. Here, the authors used LCM to spatially monitor the response to miR-21 delivery in the macrophage-enriched zones of heart postmyocardial infarction in a rodent model [47]. Multiple reports have shown that miRNAs are important regulators of pluripotency and differentiation. To unravel the function of specific miRNAs, it is important not only to analyze miRNA expression in the entire blastocyst but also to determine the site and level of expression in the inner cell mass (ICM) versus trophectoderm (TE). Using the LCM technology, it was identified that miR-155 was 50-fold highly expressed in ICM than in TE [48]. Though most of the reports have utilized LCM on cryopreserved tissue in optimal cutting temperature (OCT) compound, Majer and Booth have performed LCM on formalin-fixed FPPE brain tissue following virus infection to identify differentially expressed miRNAs [49]. The above studies utilized LCM in conjugation with RT-qPCR. With better capture techniques, LCM-captured spatial tissues are being subjected to next-generation sequencing (NGS). LCM coupled with NGS was utilized to study the specific miRNA expression profiles in the epidermis and dermal inflammatory infiltrates of psoriatic skin of patients [40]. The authors identified 24 deregulated miRNAs in the epidermis and 37 deregulated miRNAs in the dermis of psoriatic plaque compared with normal psoriatic skin. Thus, they were able to demonstrate that LCM combined with NGS provided a robust approach to explore the global miRNA expression in the epidermal and dermal compartments of the skin [40].

## 4. LCM-Based Epigenetic Studies in Skin-Related Cancer

Melanoma and nonmelanoma skin cancers such as basal cell carcinoma (BCC) and squamous cell carcinoma are the most widespread cancer in the world [50]. These cancers become even more important when it is acknowledged that sun exposure and other behaviors are the risk factors for the future development of skin cancer. With proper diagnostic steps, these cancers can be innocuous and treated. However, delayed treatment is common and is associated with increased mortality and morbidity. Early detection of suspicious skin lesions could therefore offer the opportunity for early intervention and reduced morbidity. Methylation markers for instance have been shown to be an independent prognostic indicator for skin cancer [39]. However, analysis of these markers stems on the ability to specify tissue cells of interest without unnecessary heterogeneity.

When examining melanoma in particular, a particular prognostic factor that is frequently examined is the EMT, often called mesenchymal mimicry [51]. Wouters et al. examined this transition using LCM technology to isolate melanoma cells based on their unique morphology, a task which is notoriously difficult given the large heterogeneity that exists among cutaneous cancers [51]. These cells were isolated using a Leica DM6000B and used for downstream analysis. Immunohistochemical staining was performed looking specifically for the epithelial-mesenchymal transition marker, fibronectin 1 (FN1). FN1-high melanoma cells were found to reside in hypoxic environments suggesting that there exists an interplay between environmental cues and the aggressiveness of melanoma cells [17]. Subsequently, the study raises the question about how these environmental cues affect melanoma cells.

Further elucidation of the role of epigenetic modification in cutaneous cancers is needed to understand the etiology of the disease. Cutaneous melanoma has also been a target for epigenetic investigation. In particular, the methylation status of CDKN2A and RASS1FA has been long implicated in the progression of cancer and the inactivation of p16 and p14 tumor suppressor genes [39]. However, it has also been shown that gene hypomethylation can contribute to tumorigenesis. For example, hypomethylation of long interspersed nucleotide element-1 (LINE-1) is the most studied repetitive genome-wide hypomethylation loci. Tellez et al. further linked cutaneous melanoma to increased levels of LINE-1 hypomethylation [52]. However, the largest limitation was the need for highly purified neoplastic cells. Thurin et al. outline the use of LCM (Arcturus XT) isolated purified tumor cells for downstream analysis such as qPCR or quantitative methylation specific PCR (qMSP) [39]. In their analysis, formalin-fixed paraffin-embedded tissues were cut with LCM at 5 *μ*m. The resulting tissue was prepared with hematoxylin and eosin stain, and downstream analysis confirmed the connection between LINE-1 hypomethylation and tumorigenesis.

A deeper analysis of skin samples with cutaneous T cell lymphoma (CTL) was done in 2014 and outlined the intersection between LCM and measuring DNA methylation [53]. Target biopsies from patients with CTL were able to be isolated from 5 *μ*m tissue samples via LCM. Microdissected sections were then lysed and DNA was precipitated. Promoter region *FAS* gene was amplified via PCR in place in Pyromark Q96 MD machine (GE Healthcare, Piscataway, NJ, USA) for sequencing. Pyromark Q-CpG software can then be used to analyze demethylation rates at the *FAS*-ligand promoter [53]. The authors were able to determine the level of DNA methylation of cancerous skin biopsies when compared to treatment groups receiving methotrexate.

Further analysis into cutaneous cancers showed that there exist rare prognostic markers for the poorly understood Merkel cell carcinoma (MCC) [54]. MCC has entered the forefront of investigative efforts due to its new association with the novel Merkel polyomavirus and its exploding incidence [17]. The prognosis of MCC is very poor as evident by its mortality which is over four times that of melanoma. Thus, there is a shifting focus to the possibility of early interventions to prevent this mortality. Having the information to identify MCC in an earlier, less malignant stage can also offer some preventative measures. Of note, Masterson et al. (2014) used IR-LCM and UV-LCM in combination to isolate tumor cells and identify select markers that were upregulated [37]. In particular, *mucin 1*, *KIT*, and *kinesin family member 3A* were all implicated with a poor prognosis. Epigenetic analysis of these genes would create a viable opportunity for further therapeutic interventions for MCC. The need for epigenetic analysis is ongoing, and the outlined utilization of LCM is principal.

## 5. Future Directions and Conclusion

Epigenetics has been shown to be a burgeoning field of study with huge implications in early therapeutic intervention. Of particular interest is the interplay that exists between epigenetic and environmental cues. These cues in turn are the driving force behind the extragenomic modifications as outlined in this review. Finding prognostic markers or loci of interest exists in abundance among the current literature. However, there is a noticeable dearth of information that accounts for this same approach when taking into consideration the sheer variety that certain cellular environments can have on gene expression patterns. Isolation of cells taken *in vivo* comes with the caveat that these cells will be subject to a huge array of confounding exposures that can drastically alter protein, gene, and epigenetic expression. As such with traditional capture technologies, any downstream protein or gene analysis will always run the risk of having unwanted cell types. LCM operates at this intersection. By isolating unique cells with a high degree of accuracy, LCM is able to control for the large amount of heterogeneity that is seen among *in vivo* tissue samples (Table 1). The widespread integration of LCM into laboratory settings offers an exciting new approach in the analysis of downstream cellular cascades. Cutaneous disease in particular comes into play when acknowledging the vast diversity that exists. Arguably more so than any other field of study, dermatologic pathology contains hundreds of complex cells that change rapidly based on autocrine and paracrine modifications that are heavily dependent on environmental cues. Epigenetic modifications have been shown to play a role in angiogenesis, tissue remodeling, and wound closure. These all play pivotal roles in the healing of chronic cutaneous wounds, yet another burgeoning field of interest. The application of LCM to cutaneous research has already challenged preexisting paradigms surrounding aging and cancer markers. Further investigation of these variables (such as tissue hypoxia, aging, sunlight, and carcinogen exposure) will inevitably result in different gene expression profiles than when compared to cells that were traditionally captured. In the future, larger incorporation of LCM technology can create cell samples to be used for cancer screening, regenerative medicine, and pharmacological intervention.

## Figures and Tables

**Table 1:** LCM-based studies for cutaneous manifestations.

Study and author	Disease model	Tissue element captured	Type of epigenetic modification studied
Singh et al. [42]	Diabetes	CD31^+^ endothelial element	Methylation of miR-200b promoter
Ren et al. [8]	Ovarian cancer	Tissue from ovarian carcinoma	Inactivation of hMLH1 during malignancy
Li et al. [9]	Aging	Dermal and epidermal elements	Expression of PTGES1 and COX2 mRNA expression
Wu et al. [36]	Endometriosis	Epithelial component of endometriotic implants	Methylation status of progesterone receptors (PRA, PRB)
Greenspan et al. [2]	Colon tumorigenesis	Colonic epithelial elements	Methylation status of RASSF1A
Sigalotti et al. [39]	Melanoma	Cutaneous melanoma cellular elements	Methylation of *LINE-1*
Nie et al. [41]	Chronic wounds	Diabetic ulcers	miRNA expression of NFkB, TGF-B/SMED
Singh et al. [43]	Wound angiogenesis	Human dermal microvascular endothelial cells	E-Cadherin/ZEB1 expression
Wu et al. [53]	Cutaneous T cell lymphoma	Human CTCL lines HH, Sz4, MyLa, Hut 78	CpG methylation of *FAS/CD95*
Masterson et al. [54]	Merkel cell carcinoma	Tumor resection epithelial elements	Expression of KRT20, KIF3A, and MUCI1

## Data Availability

No data were used to support this review article.
